# Model aversiveness and the evolution of imperfect Batesian mimics

**DOI:** 10.1093/beheco/arad063

**Published:** 2023-07-31

**Authors:** Thomas W Pike, Oliver H P Burman

**Affiliations:** Department of Life Sciences, University of Lincoln, Joseph Banks Laboratories, Green Lane, Lincoln LN6 7DL, UK; Department of Life Sciences, University of Lincoln, Joseph Banks Laboratories, Green Lane, Lincoln LN6 7DL, UK

**Keywords:** adaptation, Batesian mimicry, color pattern, predation

## Abstract

There are numerous examples of Batesian mimics that only imperfectly resemble their models. Given that inaccurate mimics are known to be predated more frequently than accurate ones, imperfect mimicry therefore poses something of a conundrum. One putative explanation, the relaxed selection hypothesis, predicts that when the cost of attacking a model is high relative to the benefit of consuming a mimic, selection against imperfect mimics will be relaxed, allowing mimics to be more imperfect for a given level of fitness. However, empirical support for this hypothesis is equivocal. Here, we report an experimental test of the relaxed selection hypothesis, in which human participants were tasked with discriminating between artificial stimuli representing models and mimics. In response to “attacking” a model (i.e., misclassifying it as palatable, or non-aversive) they received either a mild electric shock (high cost) or vibratory feedback (low cost). Consistent with the predictions of this hypothesis, we found that when the cost of attacking a model was high, mimetic phenotype could deviate more from the model (i.e., be more imperfect) for a given level of fitness than when the cost of attacking a model was low. Moreover, when the cost of attacking a model was high, participants showed an increased latency to attack. This finding shows that given sufficient costs, the relaxed selection hypothesis is a plausible explanation for the evolution of imperfect mimicry.

## Teaser

Batesian mimics are harmless species that resemble a protected model in order to deceive potential predators. Why some mimics only loosely resemble their models therefore poses something of a conundrum. Using a computer game involving human “predators,” we show that if models are particularly aversive (by giving a mild electric shock on capture) this can allow even poorly matched mimics to gain protection: participants not only actively avoided them but showed increased hesitancy to attack.

## INTRODUCTION

Batesian mimics are harmless species that resemble an aversive or defended model in order to deceive potential predators into misidentifying them as unpalatable or unprofitable (Bates 1862; Ruxton et al. 2004). Selection for mimicry is predicted to result in mimetic species that closely resemble their model (Ruxton et al. 2004), and some mimics and models do indeed appear (at least to human observers) almost indistinguishable (Penney et al. 2012); others, however, including some snakes (Savage and Slowinski 1992), spiders (Edmunds 2000), and hoverflies (Penney et al. 2012), share only a passing resemblance (Sherratt 2002; Gilbert 2005). The existence of imperfect mimics therefore poses something of a conundrum, particularly given that studies have repeatedly found that inaccurate mimics are predated more frequently than accurate ones (although less frequently than non-mimics) (Mostler 1935; Oaten et al. 1975; Dittrich et al. 1993; Mappes and Alatalo 1997; although see Valkonen et al. 2011; Hossie and Sherratt 2013), and a large number of hypotheses have been put forward to explain the evolution and maintenance of imperfect mimicry (reviewed in Kikuchi and Pfennig 2013; McLean et al. 2019).

**Figure 1:**
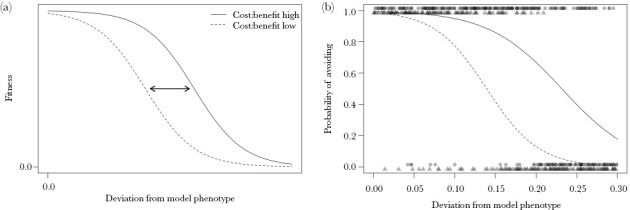
(a) Theoretical variation in fitness with respect to mimetic phenotype. When predators face a relatively high cost to attacking models (solid line), mimics can afford to be more imperfect for a given level of fitness (i.e., shifted right on the graph, away from the phenotype of their model) than when the cost of attacking models is low (dashed line). (b) The probability that participants chose to avoid a mimic when the cost of misclassification was comparatively high (i.e., they received an electric shock; solid line, circles) compared to when the cost of misclassification was low (i.e., they felt a vibration; dashed line, triangles). Data points show a participant’s decision (0, caught; 1, avoided) as a function of phenotype, where larger values indicate greater deviation from the phenotype of the model. Curves show fits from the generalized linear mixed-effects model.

The degree of model unpalatability is thought to play an important role in both the occurrence of mimicry (Lindström et al. 1997) and the evolution of mimetic phenotypes (Duncan and Sheppard 1965; Goodale and Sneddon 1977; Lindström et al. 1997). This idea is central to the relaxed selection hypothesis, which posits that the strength of selection for better mimicry should decrease as the mimic and model become increasingly similar in phenotype, and as the cost of attacking models increases (Figure 2 in Kikuchi and Pfennig 2013; see also Schmidt 1958; Duncan and Sheppard 1965; Sherratt 2002; Penney et al. 2012). This hypothesis therefore yields a clear prediction: when the cost of attacking a model is high relative to the benefit of consuming a mimic, selection against imperfect mimics will be relaxed; mimics can therefore afford to be more imperfect for a given level of fitness than when the cost:benefit ratio of attacking a model is comparatively low (Kikuchi and Pfennig 2013; Figure 1a). While this hypothesis has been widely acknowledged (Duncan and Sheppard 1965; Pilecki and O’Donald 1971; Kikuchi and Pfennig 2013), it has so far received equivocal empirical support (Goodale and Sneddon 1977; Lindström et al. 1997; Kraemer et al. 2015).

By presenting wild birds with artificial prey that varied in their phenotypic similarity to either mildly or highly distasteful models, Goodale and Sneddon (1977) provided evidence that prior experience with the highly distasteful models resulted in the rejection of mimetic prey that were less phenotypically similar to the model compared to when models were only mildly distasteful, consistent with the relaxed selection hypothesis. However, it is unclear the extent to which this was influenced by the simultaneous presentation of prey phenotypes, which is likely to be very different from the sequential encounters experienced by predators in nature (Lindström et al. 1997). To address this, Lindström et al. (1997) used two levels of bitter-tasting chloroquine solution to manipulate the level of distastefulness experienced by great tits (*Parus major*) foraging on artificial prey. They found that, as predicted, more distasteful models survived better, but the effect of model distastefulness on the survival of mimics, while in the predicted direction, did not reach statistical significance. More recently, in a correlational study on a salamander mimicry system, Kraemer et al. (2015) found no evidence that the degree of model unpalatability was related to the phenotypic similarity between model and mimic, despite considerable variation in model toxicity. The precise reasons for this are unknown, although the authors postulate that an observed positive association between body size and toxicity may have led to relaxed selection in this mimicry system, independent of variation in toxicity. Alternatively, characteristics of the predators themselves may have influenced the evolution of mimetic phenotype; for instance, limitations inherent within predator sensory systems may have prevented them from distinguishing between models and imperfect mimics, while the presence of multiple predator species may have imposed opposing selective pressures, with some predators selecting for mimicry and others against it (Kikuchi and Pfennig 2013).

Here, we provide an experimental test of the relaxed selection hypothesis, in which human participants completed a computer-based task where they had to discriminate between artificial stimuli representing models and mimics. Mimics could vary in their similarity from the average model phenotype on a continuous scale from perfect, through varying degrees of imperfect matching, while other factors (e.g., variation in model/mimic size, predator species) were held constant. Where costs to selecting a model have been imposed in similar human-based tasks elsewhere, they have been comparatively mild (e.g., a negative sound, or a decrement in a running score counter; Kikuchi et al. 2015), which may be insufficient to tease apart differences in behavior as a function of cost. To overcome this, participants in this study received either a mild electric shock (high cost) or felt a vibration (low cost) in response to “attacking” a model (i.e., misclassifying it as palatable, or non-aversive). We made the following predictions. First, that models that are more costly to attack would survive better (Lindström et al. 1997), validating our experimental manipulation of cost. Second, that when the cost of attacking a model is comparatively high, mimetic phenotypes will deviate more from the model (i.e., be more imperfect) for a given level of fitness than when the cost of attacking a model is low. And third, when the cost of attacking a model is high, the “incentive to attack”—that is, the likelihood of a predator risking an attack with an uncertain outcome (Johnstone 2002)—will be reduced, leading to more hesitancy and hence higher response latencies.

## METHODS

A total of *n* = 30 participants took part in this study, all of whom were staff or students at the University of Lincoln. Before providing consent to take part in the study, participants were given information on its general aims (although not the specific hypothesis being tested), what they would be asked to do, and the approximate time required to complete the study. The study was approved by the University of Lincoln Research Ethics Committee (reference CoSREC187).

Our general methodological approach was similar to that described by Kikuchi et al. (2015). Participants were presented with a series of colored stimuli on a computer screen, each representing either a model or a mimic, and for each trial they had to decide whether to “attack” or “avoid” the stimulus. They were informed that the overall aim of the game was to maximize their score, and that attacking a model would reduce their score by 1, while attacking a mimic would increase their score by 1. Choosing to avoid either a model or a mimic simply allowed the participant to move on to the next trial without affecting their score. They were also told that there would be a cost to attacking a model, consisting of either a mild electric shock (high cost) or a vibration (low cost). Which condition (high or low cost) a participant was allocated to was randomly determined at the start of the game, with equal probability. Both the electric shocks and vibratory feedback were delivered via a commercially available wrist band (Pavlok 2; Pavlok, Boston, MA) and was always in direct response to a participant’s action during the game (i.e., there was no experimenter intervention; if they chose to attack a stimulus there was a risk they would either receive an electric shock or feel a vibration, although if they chose to avoid a stimulus there would be no consequence). Participants were given information on the manufacturer’s health and safety guidelines, the type and frequency of the feedback they should expect (including the option of a demonstration if they wished), and what actions would result in such feedback, before agreeing to participate in the study. The electric shock was set to “1 zap” and 100% strength (approximately 450 V, 4 mA), which felt similar in type, intensity and duration to the static electric shocks received when touching a car door or shopping trolly (i.e., brief and uncomfortable, but not painful or dangerous; personal observation). The strength of the vibration was set to 100%.

**Figure 2:**
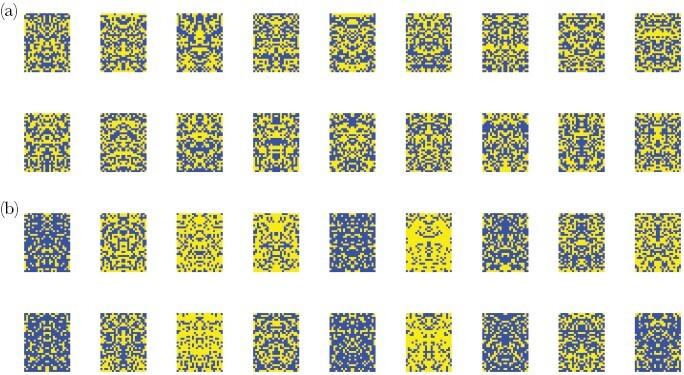
Representative examples of artificial stimuli representing (a) models, and (b) mimics. Please see text for full details.

Stimuli consisted of rectangles, 31 pixels in height × 24 pixels in width (corresponding to 9.3 × 7.2 mm on the screen), which were bilaterally symmetrical along the horizontal axis and made up of blue and yellow pixels (Kikuchi et al. 2015). The color of each pixel in the left half of the rectangle was determined randomly, with the probability of being either blue or yellow depending on whether the stimulus was designated a model or a mimic. This pattern was then mirrored on the right half of the rectangle. For models, the probability of a pixel being blue was fixed at 0.5. For mimics, this probability was drawn randomly from a uniform distribution on the interval [0.2, 0.8]; mimics could therefore exhibit a range of phenotypes, from being very model-like (i.e., having an approximately equal proportion of blue and yellow pixels) to being much bluer or much yellower (i.e., deviating from the model phenotype in either direction) (Figure 2).

At the start of a game, each participant was simultaneously shown clearly labeled exemplars of 36 randomly selected models and 36 mimics that they could study for as long as they wished before proceeding. After choosing to begin, they were then presented with a series of 30 stimuli representing either a model or a mimic, shown one at a time (Lindström et al. 1997). Each stimulus was generated using a random draw from the specified color distribution. Below the stimulus were two buttons, one labeled “Attack” and one labeled “Skip” (i.e., avoid) that allowed participants to register their decision. There was no time limit imposed on making this decision, although the decision latency (in ms) was recorded. After making their choice, the running score was updated and, if they chose to attack a model, they received the appropriate feedback (i.e., an electric shock or a vibration, depending on which condition they had been randomly assigned to). The stimulus type (model or mimic), the proportion of blue pixels in the stimulus phenotype, and the participant’s decision (attack or avoid) was recorded automatically. Each participant only played the game once.

All analyses were conducted in R 4.1.2 (R Core Team 2021). We first tested whether participants could accurately identify models, by comparing the proportion of successfully avoided models against chance levels of avoidance (a proportion of 0.5) using a binomial test. We then tested whether the probability of successfully avoiding a model differed between experimental conditions (high or low cost; i.e., electric shock or vibration, respectively) using a generalized linear mixed-effects model (with the *glmer* function from the *lme4* package; Bates et al. 2015), with attacked/avoided as the binomial response (with a logit link function), condition as a fixed factor, and participant identity as a random effect. To test for a significant difference between conditions, we compared this model to one that lacked the fixed factor using a likelihood ratio test (Crawley 2015). As a measure of effect size, we calculated the odds ratio as the observed odds (and their 95% confidence intervals) of successfully avoiding a model when in the high-cost condition relative to the low-cost condition.

We next tested whether the probability of avoiding mimics (i.e., incorrectly classifying them as models) was predicted by the interaction between the deviation in mimic phenotype from that of the model (that is, the absolute difference between the proportion of blue pixels in a given mimic and 0.5, the proportion of blue pixels in the model) and experimental condition, using a generalized linear mixed-effects model with attacked/avoided as the binomial response and participant identity as a random effect, as described above.

Finally, using the full dataset we explored the factors affecting the latency to make a decision using a linear mixed-effect model (with the *lmer* function in the *lme4* package) with log-transformed latency as the response, and experimental condition, decision (attack or avoid), and the extent of deviation from the model phenotype (and their interactions) as explanatory variables. Participant identity was included as a random effect. Post-hoc comparisons were made using similar linear mixed-effects models run on the relevant subset of the data, with *P*-values adjusted for multiple testing using a false discovery rate (FDR) correction (Benjamini and Hochberg 1995). In all cases, model assumptions were assessed via visual inspection of the normality and homoscedasticity of residuals (Gelman and Hill 2007).

## RESULTS

Models were avoided significantly more frequently than would be expected by chance (93.3% compared to 50%; binomial test: *P* < 0.001). Moreover, there was no significant effect of condition (high or low cost; i.e., electric shock or vibration, respectively) on the probability of avoidance (i.e., being correctly classified as a model) (odds ratio [95% CI]: 1.57 [0.83, 3.03], χ^2^(1) = 1.89, *P* = 0.168), suggesting that participants were equally good at identifying (and avoiding) models, regardless of the costs of misclassification.

When considering mimics, however, the probability of avoiding capture was significantly predicted by the interaction between the deviation in mimic phenotype from that of the model and experimental condition (χ^2^(1) = 5.78, *P* = 0.016), such that the deviation of a mimic’s phenotype could be greater (i.e., less model-like, more imperfect) when the cost of misclassification was relatively high than when the cost of misclassification was lower, in order to achieve the same probability of avoidance (Figure 1b). Specifically, for mimics that successfully avoided capture the deviation from the model phenotype was significantly greater (i.e., right shifted on Figure 1b) when models responded with an electric shock than when they responded with a vibration (estimate ± SE: 0.056 ± 0.008, χ^2^(1) = 36.42, *P* < 0.001). This suggests that participants were less willing to classify stimuli approaching the model in phenotype as mimics when the cost of misclassification was comparatively high.

**Figure 3:**
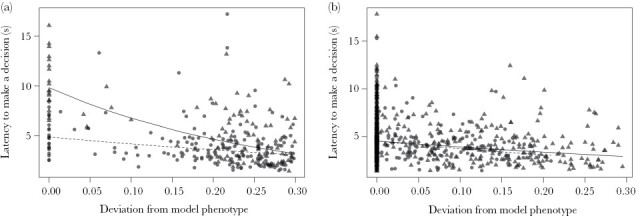
Latency to decide whether to (a) attack or (b) avoid a stimulus as a function of phenotype, where larger values indicate greater deviation from the phenotype of the model, when the cost of misclassification was either high (i.e., participants received an electric shock; triangles, solid lines) or low (i.e., they felt a vibration; circles, dashed lines).

The latency to make a decision was significantly predicted by the three-way interaction between experimental condition, whether a stimulus was attacked or avoided, and the deviation in phenotype from that of the model (χ^2^(1) = 11.11, *P* < 0.001; Figure 3). Overall, there was a significant increase in the latency to respond as a stimulus became more model-like (shown by the negative relationships evident in Figure 3a,b; estimate ± SE: −1.282 ± 0.125, χ^2^(1) = 100.71, FDR-corrected *P* < 0.001); however, the extent to which this differed between the two experimental conditions was contingent on whether or not the decision was to attack or to avoid. When only considering stimuli that were attacked, there was a significant interaction between condition and deviation from model phenotype (χ^2^(1) = 13.94, FDR-corrected *P* < 0.001) such that the disparity in response latency increased as stimuli became more model-like (Figure 3a); specifically, participants in the high-cost condition took longest to decide whether to attack stimuli that were closer to the model phenotype. In contrast, when only considering stimuli that were avoided, there was no significant interaction between condition and the deviation in phenotype (χ^2^(1) = 0.69, FDR-corrected *P* = 0.403), with participants in the both high- and low-cost conditions exhibiting comparable response latencies regardless of a stimulus’ similarity to the phenotype of the model (Figure 3b).

## DISCUSSION

A wide range of hypotheses have been put forward to explain the evolution of imperfect mimicry (reviewed in Kikuchi and Pfennig 2013; McLean et al. 2019). Here, we provide experimental evidence to support one of these mechanisms, the relaxed selection hypothesis (Schmidt 1958; Duncan and Sheppard 1965; Sherratt 2002; Penney et al. 2012), by manipulating the cost of attacking a model while holding other aspects of the prey (including, e.g., their size and relative abundance) and the type of predator (in this case, human participants) constant (Kikuchi and Pfennig 2013; Kraemer et al. 2015). Specifically, participants were tasked with discriminating between artificial stimuli representing models and mimics, where mimics could vary in their similarity to the model phenotype from perfect, through varying degrees of imperfect matching (Kikuchi et al. 2015). In response to misclassifying a model as palatable or non-aversive, participants received either a mild electric shock (a comparatively high cost) or felt a vibration (low cost), thereby ensuring that there was (at least for half the participants) a meaningful cost to this decision.

In line with previous studies, we found that attack rates decreased nonlinearly as mimics approach models in phenotype (Schmidt 1958; Duncan and Sheppard 1965; Ford 1971; Caley and Schluter 2003; Lynn et al. 2005; McGuire et al. 2006). Moreover, consistent with the predictions of the relaxed selection hypothesis (Kikuchi and Pfennig 2013), when the cost of attacking a model was comparatively high mimics could afford to be more imperfect (i.e., deviate more from the model phenotype, and hence appear less model-like) for the same level of fitness—here quantified as the probability of avoidance—than when the cost of attacking a model was lower. This relaxed selection on mimics therefore resulted in imperfect mimics that were as fit as perfect mimics/models.

There was also evidence that participants were more hesitant to make the decision to attack when they knew there was a high cost to misclassification. While overall response latencies tended to increase as a stimulus approached the model in phenotype, presumably because the discrimination became more challenging, participants took disproportionately longer to decide whether to attack a target when the cost of misclassification was high—that is, when the “incentive to attack” (Johnstone 2002) was reduced. This makes intuitive sense, since participants were specifically making a decision that could result in a mildly painful electric shock. Indeed, one possible reason why much of the empirical evidence supporting relaxed selection on mimics when models are more toxic has so far been equivocal (e.g., Lindström et al. 1997; Kraemer et al. 2015) may be because the costs involved were not sufficient. For example, birds are known to tolerate bitter tasting artificial prey (Skelhorn and Rowe 2010) whereas the potential cost of attacking a real model can be substantial: wasp stings, for instance, while not necessarily fatal to birds (although see Phisalix 1935; Fry 1969) can cause severe discomfort and may interfere with activities such as foraging (Birkhead 1974), while some ladybird species (Coccinellidae) are known to be highly toxic to blue tits (*Cyanistes caeruleus*) (Marples et al. 1989). Sufficiently severe costs may therefore be important for the evolution of imperfect mimicry via relaxed selection, not only via misclassification and avoidance but also by affording an increased opportunity to escape through the increased hesitancy to attack (Bosque et al. 2018) or by reducing the time available for predators to search for prey in the future (Abbot and Sherratt 2013).

In summary, while there are numerous explanations for the evolution of imperfect mimicry, each of which is likely to be specific to a particular ecological context (Kikuchi and Pfennig 2013), here we provide convincing evidence that when there is a sufficiently high cost to attacking a model, selection on mimics is relaxed. This results in imperfect mimics that are as fit as perfect mimics, consistent with the predictions of the relaxed selection hypothesis (Duncan and Sheppard 1965; Pilecki and O’Donald 1971; Goodale and Sneddon 1977; Lindström et al. 1997; Kikuchi and Pfennig 2013; Kraemer et al. 2015).

## Data Availability

Analyses reported in this article can be reproduced using the data provided by Pike and Burman (2023).
